# Protein Hydroxylation Catalyzed by 2-Oxoglutarate-dependent Oxygenases*

**DOI:** 10.1074/jbc.R115.662627

**Published:** 2015-07-07

**Authors:** Suzana Markolovic, Sarah E. Wilkins, Christopher J. Schofield

**Affiliations:** From the Chemistry Research Laboratory, University of Oxford, Mansfield Road, Oxford, OX1 3TA, United Kingdom

**Keywords:** chemical biology, dioxygenase, enzyme catalysis, gene expression, hydroxylase, post-translational modification (PTM), protein chemical modification, protein synthesis, 2-oxoglutarate, hydroxylation

## Abstract

The post-translational hydroxylation of prolyl and lysyl residues, as catalyzed by 2-oxoglutarate (2OG)-dependent oxygenases, was first identified in collagen biosynthesis. 2OG oxygenases also catalyze prolyl and asparaginyl hydroxylation of the hypoxia-inducible factors that play important roles in the adaptive response to hypoxia. Subsequently, they have been shown to catalyze *N*-demethylation (via hydroxylation) of *N*^ϵ^-methylated histone lysyl residues, as well as hydroxylation of multiple other residues. Recent work has identified roles for 2OG oxygenases in the modification of translation-associated proteins, which in some cases appears to be conserved from microorganisms through to humans. Here we give an overview of protein hydroxylation catalyzed by 2OG oxygenases, focusing on recent discoveries.

## Introduction

The ferrous iron and 2-oxoglutarate (2OG)^4^-dependent oxygenases were first identified as playing roles in the post-translational modification of collagen, where they catalyze C-3 and C-4 prolyl and C-5 lysyl hydroxylations (1–3). Subsequently, 2OG oxygenases and related enzymes have been found to have multiple other biological roles and, at least in plants and microbes, to catalyze a remarkably wide range of oxidative reactions (4). In animals, the identified reactions catalyzed by 2OG oxygenases are at present limited to hydroxylation (sometimes also including sequential oxidation of the resulting alcohols into aldehydes and acids) and demethylation of *N*-methylated groups in proteins and nucleic acids, which likely occurs via initial hydroxylation of the methyl group. Although the addition and removal of hydroxyl and methyl groups to proteins represent small and chemically neutral post-translational modifications, they can in some cases have profound biological effects. Indeed, several 2OG oxygenases catalyzing protein hydroxylation are current chemotherapeutic targets (5).

**FIGURE 1.**
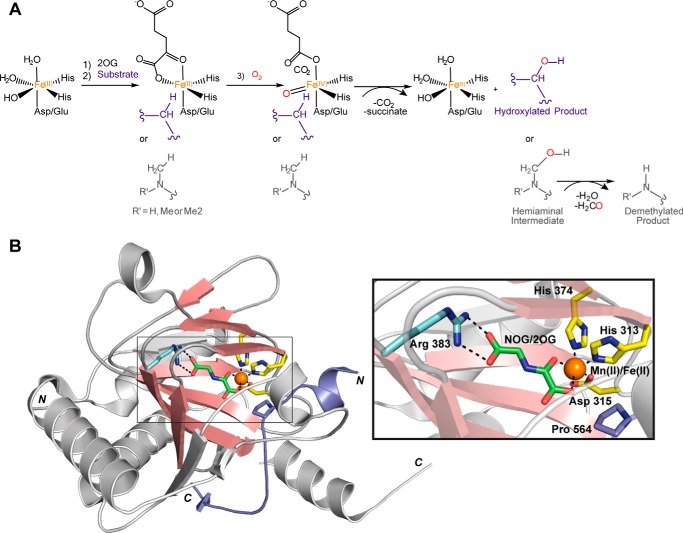
**Outline mechanism and characteristic fold of 2OG oxygenases.** *A*, within the active site, Fe(II) (*orange*) is bound in an octahedral manner by an H*X*(D/E) … H facial triad. The remaining coordination sites are initially occupied by water molecules, two of which are displaced upon binding of 2OG. Binding of the substrate (*purple*) displaces the final water molecule, creating an open coordination site for oxygen (*red*) to bind. Oxidative decarboxylation of 2OG generates succinate and CO_2_ and yields a ferryl intermediate (Fe^IV^=O) that reacts with the substrate to form the hydroxylated product. Note that *N*-methyl demethylation (shown in *gray*) occurs via hydroxylation of the methyl group to form a hemiaminal intermediate that subsequently collapses to yield formaldehyde and the demethylated product. *B*, view from a crystal structure of a PHD in complex with a HIF-1α fragment peptide substrate (Protein Data Bank ID: 3HQR). The core double-stranded β-helix fold (*salmon*) is conserved in 2OG oxygenases and consists of eight β-strands that form two anti-parallel β-sheets. The HIF-1α peptide (*purple*) binds such that the side chain of Pro 564 is oriented toward the metal within the active site. A magnified view of the active site (*right*) highlights residues involved in binding Fe(II) (*yellow*) and 2OG (*light blue*). Note that in this structure, *N*-oxalylglycine (*NOG*) and Mn(II) replace 2OG and Fe(II), respectively.

In addition to the roles associated with protein modification, 2OG oxygenases function in fatty acid metabolism, carnitine biosynthesis, and phytanic acid catabolism, as well as in DNA and mRNA repair, regulation, and modification (6). 2OG oxygenases employ a conserved mechanism in which sequential binding of 2OG to the active site is followed by that of substrate and then oxygen (4, 7). Oxidative decarboxylation of 2OG yields a ferryl intermediate (Fe^IV^=O), which reacts with the substrate to effect 2-electron oxidation, normally hydroxylation (Fig. 1*A*). *N*-Methyl demethylation proceeds via initial hydroxylation of the methyl group to form a hemiaminal intermediate, which fragments to give formaldehyde and the demethylated product.

Extensive structural studies have revealed that the catalytic domains of 2OG oxygenases have a conserved core fold comprising a distorted double-stranded β-helix (also known as a jelly-roll, cupin, or Jumonji-C (JmjC) fold) that supports conserved binding motifs for ferrous iron and 2OG (Fig. 1*B*) (8). The iron is normally complexed by three protein residues comprising an H*X*(D/E) … H motif, although there are variations on this motif, including in potential “pseudo-enzymes.” The mode of 2OG binding involves electrostatic interactions between the C-5 carboxylate of 2OG and a basic lysyl or arginyl residue and normally one alcohol side chain (9). The 2OG binds to the iron in a bidentate manner, leaving one site free for water/oxygen binding. Substrate binding promotes release of the water from the metal, thus promoting oxygen binding (4). There is greater variation in the mode of substrate binding when compared with those of Fe(II) or 2OG; substrate binding can induce substantial conformational changes, which may be particularly important in the case of macromolecular substrates such as proteins. As yet, there are few studies on the conformational changes involved in binding of full-length protein substrates by 2OG oxygenases; a recent study of a prokaryotic prolyl hydroxylase in complex with its substrate illustrates the potential for large conformational changes in both 2OG oxygenase and substrate during binding (10). Further, most 2OG oxygenases acting on proteins have additional “non-catalytic” binding domains. Thus, at least in some cases, 2OG protein hydroxylases may be best viewed as modulating protein-protein interactions in a manner in which the catalytic modification plays a role, but which is not necessarily a sole end in itself.

The discovery in 2001 that hydroxylation can play physiologically relevant roles in transcriptional regulation has stimulated work on the function of the ∼60 or so human 2OG oxygenases (11–13). This work has identified new roles for these enzymes in protein and nucleic acid modifications and revealed that they likely play roles in all stages of protein biosynthesis in animals, *i.e.* at transcriptional, splicing, and translational levels. Protein hydroxylations catalyzed by human 2OG oxygenases are summarized in Table 1. It should be noted that there are other examples of protein hydroxylases beyond the scope of this review that do not use 2OG as a substrate and that are structurally unrelated to the 2OG oxygenases (see *e.g.* Ref. 14). The main purpose of this minireview is to introduce the non-expert to the expanding role of 2OG oxygenases, focusing on protein oxidation; where appropriate we direct the reader to specialist reviews.

**TABLE 1:**
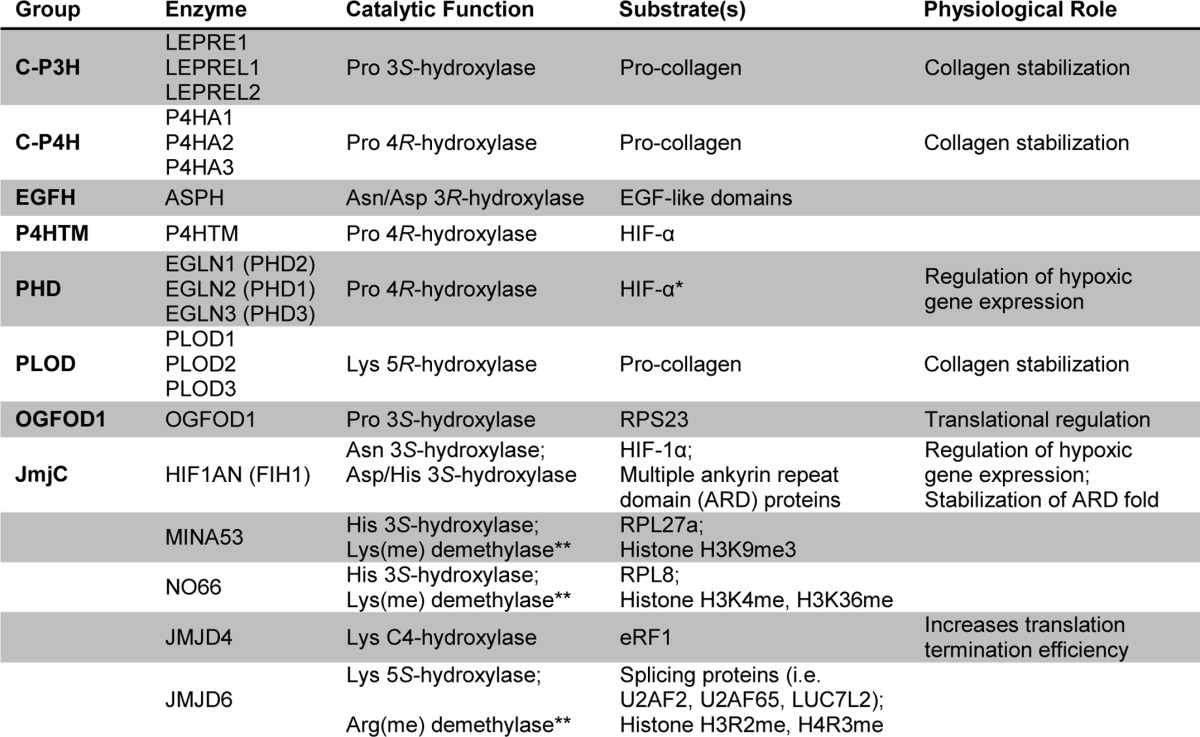
**Human 2OG oxygenases catalyzing protein hydroxylation**

* Other hydroxylation substrates for the PHDs have been reviewed elsewhere (58).

** Controversial functional assignment.

## Collagen Hydroxylases

There are three types of 2OG oxygenase with roles in collagen biosynthesis: the C-3 and C-4 prolyl hydroxylases and the C-5 lysyl hydroxylases, all of which catalyze modifications of the pro-collagen polypeptide in the endoplasmic reticulum (1–3). C-4 prolyl hydroxylation of multiple residues in the *Y*-position of Gly-*X-Y* motifs in collagen stabilizes the collagen triple helical fold (15). C-3 prolyl hydroxylation is less abundant in collagen and occurs subsequent to C-4 prolyl hydroxylation (16). The molecular roles of collagen C-3 prolyl hydroxylation are unclear, although it is proposed to cause local (de)stabilization of the triple helix, thus enabling cross-linking (17). Nevertheless, it is clearly of biological importance as evidenced by animal studies and diseases associated with a reduced level of such hydroxylation (18). Like C-4 prolyl hydroxylation, collagen C-5 lysyl hydroxylation occurs at the *Y*-position of Gly-*X-Y* motifs (19). Lysyl hydroxylation enables glycosylation (20) and, after further oxidation of the *N*^ϵ^-amino group by an amine oxidase, cross-linking (21, 22).

The roles of 2OG oxygenases in extracellular protein modifications are likely not fully defined. Recent work has identified a new type of cross-link involving reaction of C-5 hydroxylysine and methionine residues on adjacent proteins to give a sulfilimine (-S=N-) link (23). The primary role of, and apparent selectivity for hydroxylysine in this reaction is unclear, but C-5 hydroxylation may stabilize the sulfilimine link. Overall, the work on collagen hydroxylases is illustrative of the challenges of functional assignment of 2OG oxygenases. The link between the biochemical and biological roles of C-4 prolyl hydroxylation in stabilizing the collagen triple helix is unusually clear cut; in the case of C-3 prolyl and C-5 lysyl hydroxylation, the links between biochemistry and biology are much less clear.

## EGF-like Domain Hydroxylation

The first evidence that non-collagen/collagen-like proteins undergo hydroxylation came with the identification of C-3 hydroxylation of aspartyl and asparaginyl residues in EGF-like domains (24, 25). This work indicated that multiple proteins might be hydroxylated including coagulation factors (VII, IX, and X), protein C, complementation factors, thrombomodulin, the low density lipoprotein receptor, and Notch ligands. These hydroxylations are catalyzed by aspartyl/asparaginyl β-hydroxylase (ASPH), which localizes to the endoplasmic reticulum (26). Emerging structural results support the proposal that ASPH (of which there are >12 human splice variants) has an unusual active site in that it only has two protein-bound metal ligands, as it lacks the D/E residue of the typical H*X*(D/E) … H iron binding motif (27). The ASPH work is important in that it revealed that a single 2OG oxygenase can catalyze the hydroxylation of multiple protein substrates; such promiscuity is also manifested by at least one other human/animal 2OG oxygenase, factor inhibiting HIF (FIH) (see below). Like many of the FIH-catalyzed hydroxylations, the role of ASPH-catalyzed hydroxylation is unclear. Some of the ASPH-hydroxylated aspartyl residues are involved in calcium binding, although NMR studies indicate that C-3 aspartyl hydroxylation does not substantially alter calcium binding (28). Despite an undefined biochemical role for ASPH-catalyzed hydroxylation, mutations in *ASPH* are associated with severe facial abnormalities (27), and *ASPH* overexpression is linked to malignant transformation and poor prognosis in human cancers (29). ASPH knock-out mice display severe developmental phenotypes including palate defects and syndactyly; some of this may relate to ASPH catalysis in Notch-mediated signaling, although how is unclear (30).

## 2OG Oxygenases in Hypoxia Sensing

A breakthrough in functional assignment of the human 2OG oxygenases, especially with respect to roles in signaling, came with the identification of two types of 2OG oxygenase involved in the hypoxic response (11, 12, 31–33). The hypoxic response in animals involves up-regulation of the α-subunit of the α,β-hypoxia-inducible transcription factor (HIF), which regulates the expression of hundreds of genes, the precise set of which is context-dependent (34). The intact α,β-HIF transcription factor binds to hypoxic response elements associated with target genes, as first shown by work with the erythropoietin (*EPO*) promoter (35). HIF target genes include those that promote increased oxygen supply to hypoxic tissues (*e.g. EPO* and *VEGF*) and those enabling a metabolic shift toward aerobic glycolysis (Warburg effect), which is proposed to conserve oxygen use (36).

2OG oxygenases play key roles in the hypoxia-sensing mechanism of the HIF system. In humans, three prolyl hydroxylase domain (PHD) enzymes catalyze C-4 prolyl hydroxylation in two regions of HIF-α isoforms: the N- and C-terminal oxygen-dependent degradation domains (11, 12, 31, 37). This modification substantially increases (∼1000-fold) the affinity of HIF-α for the von Hippel-Lindau protein (38), which is the substrate-targeting component of an E3 ubiquitin ligase complex, and thus increases signaling for HIF-α degradation (39, 40). HIF-α prolyl hydroxylation and degradation are highly efficient, such that in normal (normoxic) cells, HIF-α is barely detectable. As oxygen levels decrease, PHD activity decreases, thus enabling HIF-α levels to rise and the hypoxic response to be “switched on.” There is substantial evidence arising from genetic and biochemical studies that the PHDs act as important hypoxia sensors for the HIF system (41). PHD activity is sometimes limited by iron and 2OG availability, at least in an *in vitro* context (42). Nonetheless, biochemical and cellular studies indicate that PHD2 (the most important of the human PHDs) is unusually sensitive to changes in oxygen availability, consistent with its proposed role as a hypoxia/oxygen sensor (43–45).

A second type of 2OG oxygenase, FIH also acts on HIF-α subunits (32, 33). FIH catalyzes C-3 hydroxylation of an asparaginyl residue in the C-terminal transactivation domain of HIF-α, a modification that substantially reduces the otherwise tight binding of HIF-α to the CREB-binding protein (CBP)/p300 transcriptional co-activator proteins (13). Thus, in contrast to PHD-catalyzed HIF-α hydroxylation, which “makes” a protein-protein interaction, that of FIH “breaks” a protein-protein interaction. Interestingly, isolated recombinant FIH is less sensitive than PHD2 to oxygen availability, a property that is reflected in the cellular activities of FIH and the PHDs, with the former being more active under hypoxic conditions (46, 47). Thus, the PHDs are likely more important than FIH in terms of their hypoxia-sensing capacity; this property has, together with the discovery of alternative substrates for FIH (see below), led to the PHDs being the preferred target for pharmaceutical activation of HIF-mediated transcription. PHD inhibitors are currently in late stage clinical trials for the treatment of anemia via up-regulation of EPO (48). PHD-like enzymes have been identified in early animals; in *Dictyostelium discoideum* (which does not contain HIF), a PHD homologue catalyzes the C-4 prolyl hydroxylation of the SKP1 subunit of an E3 ligase, a modification that enables subsequent glycosylation and that is proposed to act in a hypoxia-sensing capacity (49). The recent identification of a PHD homologue in *Pseudomonas* spp. suggests prokaryotic origins for the animal prolyl hydroxylases (10).

2OG oxygenases are classed into subfamilies based on sequence similarities within their double-stranded β-helix domain (50). The PHDs are part of the same structural subfamily as the collagen prolyl hydroxylases, whereas FIH is a member of the JmjC subfamily (51). Notably, FIH was the first JmjC protein shown to have activity as a 2OG oxygenase (33). The distinct biochemical properties of FIH and the PHDs are reflected in their structures (52–56). Unlike PHD2, FIH is dimeric and binds 2OG in a different manner and in a larger pocket than the PHDs. Conformational changes are induced in substrate binding by both FIH and the PHDs, although current evidence indicates that they are more profound for the PHDs.

Alternative substrates for both the PHDs and FIH have been reported (57, 58), although in neither case has the physiological relevance of the hydroxylation of these potential alternative substrates been established. Here we limit description to FIH because of the range of alternative substrates described. As supported by studies in animals and cells, FIH has been shown to accept multiple substrates from the ankyrin repeat domain (ARD) structural family, including Notch, transcription factors, ion channels, and cytoskeletal ARD proteins (57). Protein analysis reveals that FIH interacts with multiple ARD proteins, not all of which undergo hydroxylation (59, 60). In contrast to HIF-α hydroxylation or collagen C-4 prolyl hydroxylation, ARD hydroxylation is inefficient, ranging from 0 to 80% (61). The role of FIH-catalyzed ARD hydroxylation is unclear; in some cases it can stabilize the ARD fold, but the effect is much less than, for example, that of C-4 prolyl hydroxylation on the stability of the collagen triple helix (60). It is proposed that competition between ARDs and HIF-α for FIH can modulate the role of FIH in the hypoxic response (62). Because hydroxylated ARDs bind less tightly to FIH than unhydroxylated ARDs, ARD hydroxylation has the potential to enable a “memory” effect of hypoxic events (63). Nonetheless, unlike HIF-α prolyl and asparaginyl hydroxylations, as yet no “switch-like” roles for ARD hydroxylation have been identified, as is the case for most 2OG oxygenase-catalyzed protein hydroxylations. From a biochemical perspective, FIH-catalyzed ARD hydroxylation is interesting not only because the canonical ARD structure must unfold in order for FIH to catalyze hydroxylation (62), but also because FIH can also hydroxylate histidinyl (as in tankyrase-2) and aspartyl (as in AnkyrinR) residues in addition to its normally preferred asparaginyl residue substrates (64, 65). The substrate scope of purified recombinant FIH is even wider (66). Thus, FIH is a highly promiscuous oxygenase, a property likely shared by some other 2OG oxygenases acting on proteins (see below).

## JmjC Histone Demethylases

Post-translational modifications to the tails of histone proteins play central roles in the regulation of gene expression in eukaryotes (67). Methylation of the *N*^ϵ^-amino group of lysyl residues, especially in the histone H3 N-terminal tail, is well established as a regulatory mechanism. Unlike lysine *N*^ϵ^-acetylation, which is transcriptionally activating, *N*^ϵ^-methylation can be either transcriptionally activating or repressive depending on the context. Although the evidence for demethylation of histones goes back decades (68), it is only relatively recently that the enzymes which catalyze demethylation have been identified and characterized (69–71). The JmjC subfamily of 2OG oxygenases comprises the largest identified family of lysine demethylases (KDMs) with ∼15 human members, but its members were identified after the discovery of the flavin-dependent lysine-specific demethylases (LSDs (69)). The LSDs apparently operate via a mechanism analogous to other flavin-dependent oxidases, which limits their substrate scope to di- and mono-methylated forms of *N*^ϵ^-methylated lysyl residues. In contrast, the JmjC KDMs work via the consensus 2OG oxygenase mechanism to give a hemiaminal intermediate, which likely spontaneously collapses to give the demethylated product and formaldehyde (72). Evidence for this mechanism comes from the direct and indirect observation of formaldehyde production and from the use of substrate analogues, some of which react with JmjC KDMs to give stable alcohol products (73). It is notable that the JmjC KDMs can act on methyl groups attached to a positively charged nitrogen (which must be the case for the trimethylated state), further illustrating the catalytic potential of 2OG oxygenases. In contrast, the LSDs are proposed to operate (at least in terms of the oxidation step) on the neutral form of their substrates.

Protein *N*^ϵ^-lysine methylation is a common modification, and there is accumulating evidence that JmjC KDMs may act on non-histone substrates (74). Although the only identified biologically relevant reactions catalyzed by the JmjC KDMs are demethylations, several studies show that they have the potential to catalyze other types of dealkylation/other reactions (73), as is the case with some 2OG oxygenases acting on nucleic acids. Further, likely all or near all JmjC KDMs have additional non-catalytic domains that are of major functional relevance (51). This is beautifully exemplified in the case of PHF8 (KDM7B), where the catalytic JmjC domain is guided to its histone H3 dimethyl-lysine 9 (K9me2) substrate by the interaction of an adjacent plant homeobox domain, which latches onto the histone H3 K4me3 modification, as shown by crystallographic and other biophysical analyses (75). Thus, although there is evidence that context-dependent methylation/demethylation events are important regulatory processes in transcriptional regulation, it also seems likely that the kinetics of the non-covalent protein-protein interactions play equally if not more important roles, although these are considerably more difficult to qualify in a cellular context. Indeed, the lack of methods for qualitatively analyzing demethylation is hindering functional assignments, especially of non-histone substrates. It is also notable that some JmjC KDMs appear to lack some metal binding moieties and are likely pseudo-enzymes.

## JMJD6

**FIGURE 2.**
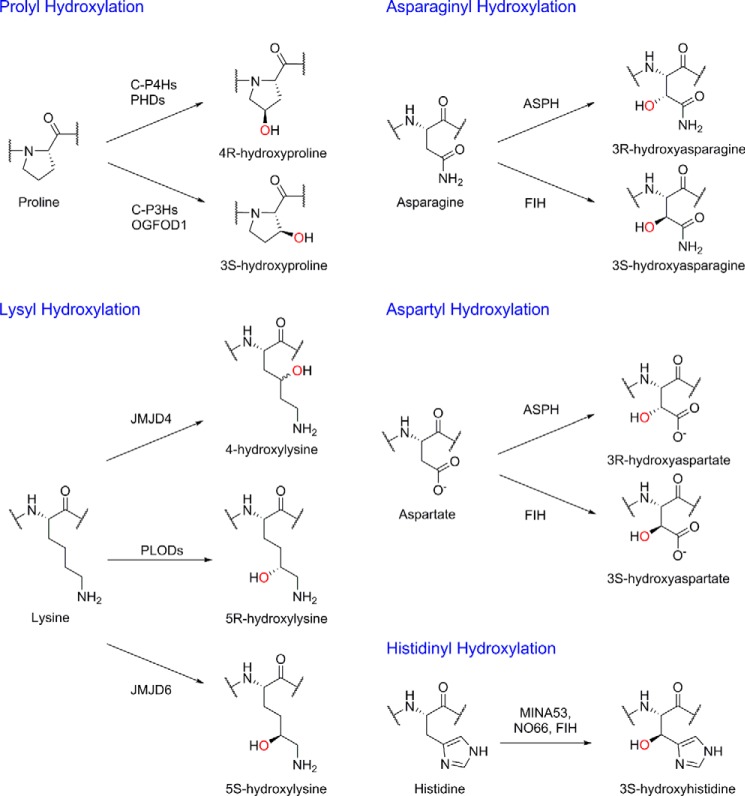
**Hydroxylations catalyzed by human 2OG oxygenases.** Human 2OG oxygenases catalyze the stereoselective hydroxylation of prolyl, lysyl, asparaginyl, aspartyl, and histidinyl residues in protein substrates; the oxygen atom incorporated into the product is shown in *red*. Note that the stereochemistry of JMJD4-catalyzed hydroxylation is unknown. *C-P4Hs*, collagen prolyl 4-hydroxylases; *C-P3Hs*, collagen prolyl 3-hydroxylases; *PLODs*, pro-collagen lysine 2-oxoglutarate 5-dioxygenase enzymes.

The JmjC subfamily of 2OG oxygenases contains both hydroxylases, such as FIH, as well as demethylases (51). This dual functionality has led to some controversy in the literature regarding functional assignment, which is well illustrated by the case of JMJD6. JMJD6 (as it is now known) was first assigned as having a key role in apoptosis, acting as a membrane-associated phosphatidyl serine receptor (76, 77). However, this assignment now seems unlikely to be correct; JMJD6 is a 2OG oxygenase that predominantly localizes to the nucleus. JMJD6 was then reported as a 2OG oxygenase acting on *N*-methylated arginine residues in histone H3, a result that if correct represents the first biochemical evidence for direct removal of arginyl methylation (78). However, subsequent work using NMR and MS analysis of products formed by purified recombinant JMJD6 has shown JMJD6 to be a lysyl C-5 hydroxylase (79); evidence for such activity was also present in an initial report of JMJD6 as an arginyl demethylase (78). Conflicting studies have continued to appear regarding the catalytic activity of JMJD6, leading to the possibility that it has dual functionality. Nonetheless, although we are somewhat biased, there is clear evidence that JMJD6 acts as a lysyl C-5 hydroxylase, interestingly giving the 5*S*- rather than the 5*R*- stereochemistry, and thus contrasting with the pro-collagen lysyl hydroxylases (80) (Fig. 2). The evidence for demethylation of *N*-methylated arginyl residues is much weaker, and there is no evidence for JMJD6 acting as a KDM.

The situation with respect to the substrates that JMJD6 acts on is also complex. JMJD6 can act on histones (as a hydroxylase and, maybe as an arginyl demethylase), although whether this activity directly regulates transcription is unclear (81). Recent work has identified a role for JMJD6 in regulating transcriptional pause release (82); this study also reported supporting evidence for the controversial histone H4 arginyl demethylation as reported by Chang *et al.* (78). JMJD6 has been reported to interact with, and at least under *in vitro* conditions, catalyze the hydroxylation of lysyl residues in splicing-associated proteins, as first shown by work on U2AF65 (79). This led to the possibility that JMJD6 regulates mRNA splicing, a hypothesis supported by work with model systems. There is evidence that JMJD6 can regulate splicing of the VEGF receptor, potentially in a manner regulated by hypoxia and/or Fe(II) availability (83).

The JMJD6 story is far from complete; there is evidence that it accepts multiple splicing-related proteins as substrates and interacts with others it does not hydroxylate (84). Further, it interacts with RNA (85) and can undergo auto-hydroxylation (86), and its localization (and hence likely activity) is regulated by oligomerization (87). Thus, like FIH, but with an even greater level of complexity, JMJD6 appears to be promiscuous, possibly having multiple roles, although some may be more physiologically relevant than others. Overall, the JMJD6 story to date illustrates the difficulty in making secure functional assignments with oxygenases that likely have multiple roles; such assignments require combined biochemical, cellular, and whole animal studies.

## Ribosomal Oxygenases and JmjC Hydroxylases

A growing body of work suggests that 2OG oxygenases are widespread regulators of translation. The first evidence for this came from the discovery of two nucleic acid oxygenases, TYW5 and ALKBH8, that catalyze hydroxylation of bases at the “wobble” position of amino-acyl tRNAs: tRNA^Phe^ and tRNA^Gly^, respectively (88, 89). Recent studies have built on these findings and shown that several ribosomal proteins and translation elongation/release factors are also 2OG oxygenase substrates. To date, three human 2OG oxygenases: MINA53, NO66, and OGFOD1, have been assigned as “ribosomal oxygenases” (ROXs). MINA53 and NO66 catalyze C-3 hydroxylation of histidinyl residues in Rpl27a and Rpl8, respectively (90). Like JMJD6, they belong to the JmjC subfamily and were originally assigned as histone demethylases acting on tri- and mono-methylated lysyl residues in histones H3 and H4. However, biochemical and MS analyses support their assignment as protein hydroxylases rather than demethylases (90). The side chains of the target histidinyl residues extend into the ribosome core, and in the case of Rpl8, toward the peptidyl transferase center. Although the biological consequences of these hydroxylation events remain to be defined, an effect on translation is consistent with the cellular roles of MINA53 and NO66 in regulating growth and proliferation (91, 92).

OGFOD1 catalyzes C-3 prolyl hydroxylation of RPS23, a modification conserved in eukaryotes ranging from yeasts to humans (93–95). The hydroxylated prolyl residue is located within the ribosome decoding center; interestingly, hydroxylation at this position has a profound effect on stop codon recognition in yeast. Tpa1, the *Saccharomyces cerevisiae* OGFOD1 homolog, catalyzes two sequential hydroxylations on the same prolyl residue to give dihydroxyproline in Rps23p (94); however, the functional significance of the second hydroxylation is unclear. Further work is required to understand the effects of OGFOD1 on protein synthesis in human cells, as well as the extent to which these are mediated by RPS23 hydroxylation.

Beyond the hydroxylation of ribosomal proteins, 2OG oxygenases influence translation via the hydroxylation of ribosome-associated proteins. The eukaryotic release factor eRF1 undergoes lysyl hydroxylation, as catalyzed by JMJD4 (96). In contrast to JMJD6 and collagen lysyl hydroxylases, JMJD4 catalyzes C-4 lysyl hydroxylation (Fig. 2). eRF1 is involved in translation termination; it recognizes stop codons as they enter the ribosomal A site and, together with the GTPase eRF3a, triggers release of the nascent polypeptide chain and ribosome disassembly. The JMJD4-hydroxylated lysyl residue lies in a conserved NIKS motif, essential for stop codon recognition; JMJD4-catalyzed hydroxylation of eRF1 increases translation termination efficiency (96).

ROXs are not only present in eukaryotes; an *Escherichia coli* homologue of MINA53 and NO66, ycfD, catalyzes C-3 hydroxylation of an arginyl residue in ribosomal protein L-16 (90). L-16 is an essential component of the bacterial ribosome, required for ribosome assembly, aminoacyl tRNA binding, and efficient peptidyl tRNA hydrolysis (97). Depletion of the *ycfD* gene in *E. coli* leads to growth retardation accompanied by a reduction in global translation rate (90). Crystallographic studies reveal that ycfD is structurally similar to human JmjC proteins, with a conserved jelly-roll fold as well as motifs for binding 2OG and Fe(II) (98, 99). This is interesting from an evolutionary perspective, as it suggests that 2OG oxygenases, in particular those that catalyze protein hydroxylation, may have prokaryotic origins. This notion is further supported by the discovery of a prolyl hydroxylase in *Pseudomonas* spp. (PPHD), which is related to (and likely an early ancestor of) the HIF prolyl hydroxylases (10). PPHD catalyzes prolyl hydroxylation of the translation elongation factor EF-Tu. Although the functional effect of hydroxylation has yet to be determined, this work provides evidence to support a conserved role for 2OG oxygenases in translational regulation.

Taken together, these data suggest that the cellular protein biosynthesis machinery is a common target for 2OG oxygenase-catalyzed hydroxylation. Further functional characterization of the human ROXs will be essential to fully understand their effects on translation and how these relate to their roles in cellular growth, proliferation, and cancer.

## Summary and Future Perspectives

**FIGURE 3.**
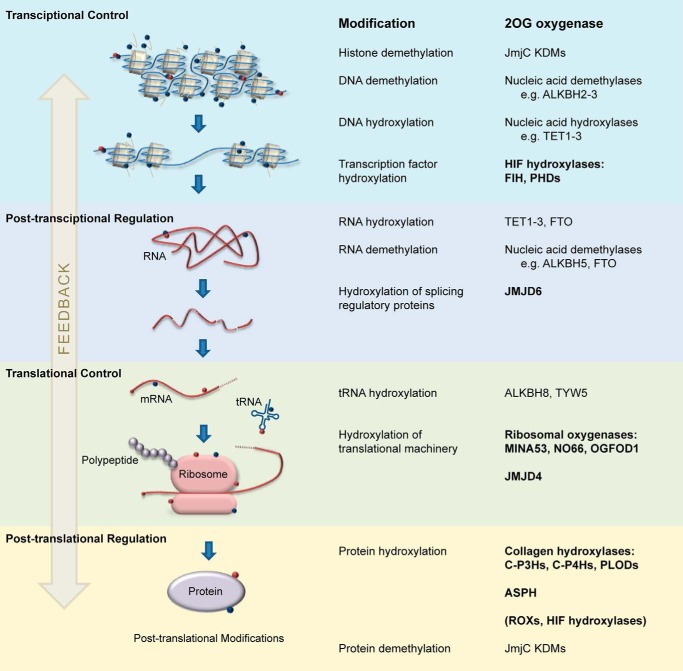
**2OG oxygenases involved in protein synthesis.** 2OG oxygenases catalyze hydroxylation and demethylation reactions that regulate transcriptional, post-transcriptional, translational, and post-translational processes. The names of enzymes that catalyze hydroxylation are in *bold. ALKBH*, alkylated DNA repair protein alkB homolog; *TET1–3*, ten-eleven translocation 1–3; *FTO*, fat mass- and obesity-associated protein; *TYW5*, tRNA wybutosine-synthesizing protein 5; *C-P4Hs*, collagen prolyl 4-hydroxylases; *C-P3Hs*, collagen prolyl 3-hydroxylases; *PLODs*, pro-collagen lysine 2-oxoglutarate 5-dioxygenase enzymes; *P4HTM*, transmembrane prolyl 4-hydroxylase.

The past 15 years have seen major advances in our understanding of the extent of 2OG oxygenase-catalyzed modifications to proteins. We now know that such modifications are likely common in all eukaryotes and in many prokaryotes, but not archaea. 2OG oxygenase-catalyzed post-translational modifications to proteins, along with related modifications to nucleic acids, are involved in all stages of protein biosynthesis in animals (Fig. 3). Although controversies remain and much work is still to be done, one can start to envisage how combined biochemical and cellular approaches will lead to the assignment of molecular functions for all human 2OG oxygenases, *i.e.* defining the reactions they catalyze and the substrate(s) that they accept. However, for only a few of the identified modifications are the cellular roles linked to physiology. Notably, these include the pioneering discoveries of collagen C-4 prolyl hydroxylation, HIF-α prolyl hydroxylation, and in some cases, histone demethylation. Future work, guided by genetic analyses, can now be focused on this objective.

## Author Contributions

All authors contributed to the writing and editing of this manuscript.

## Footnotes

4 The abbreviations used are:

2OG: 2-oxoglutarate
ARD: ankyrin repeat domain
ASPH: aspartyl/asparaginyl β-hydroxylase
EPO: erythropoietin
FIH: factor inhibiting HIF
HIF: hypoxia-inducible factor
JmjC: Jumonji-C
JMJD4/6: Jumonji domain-containing 4/6
KDM: lysine demethylase
LSD: flavin-dependent lysine-specific demethylase
MINA53: MYC-induced nuclear antigen 53
NO66: nucleolar protein 66
OGFOD1: 2-oxoglutarate and iron-dependent oxygenase domain containing 1
PHD: prolyl hydroxylase domain-containing
ROX: ribosomal oxygenase
CREB: cAMP-response element-binding protein.
